# Multi-Level Resources to Support Health Behavior Maintenance

**DOI:** 10.1093/geroni/igaa057.3022

**Published:** 2020-12-16

**Authors:** Susan Hughes, Janet Bettger, Mina Raj, Jaime Hughes

**Affiliations:** 1 University of Illinois at Chicago, Chicago, Illinois, United States; 2 Duke University School of Medicine, Durham, North Carolina, United States; 3 University of Michigan, Ann Arbor, Michigan, United States

## Abstract

Although behavior change is largely thought to occur on the individual level, maintenance of health behaviors also depends upon factors at the community and population levels. As discussed in earlier presentations, health behaviors can be influenced by social support, environmental context, and population-level policies. For example, maintaining a physical activity regimen is made easier when an older adult has access to a safe environment, support of family and friends, and/or ongoing access to a program that is continuously offered, or sustained, at a local community center. This presentation will focus on the importance of discussing sustainability when considering long-term maintenance of health behaviors. Specifically, this presentation will use examples from the evidence-based Fit & Strong! program, now being offered in 32 states, to explore sustainability of programs at the participant and community level and will provide an overview of barriers and facilitators faced by community organizations.

